# Dimethyl fumarate is highly cytotoxic in KRAS mutated cancer cells but spares non-tumorigenic cells

**DOI:** 10.18632/oncotarget.24144

**Published:** 2018-01-10

**Authors:** Nathaniel Edward Bennett Saidu, Marie Bretagne, Audrey Lupo Mansuet, Pierre-Alexandre Just, Karen Leroy, Olivier Cerles, Sandrine Chouzenoux, Carole Nicco, Diane Damotte, Marco Alifano, Bruno Borghese, François Goldwasser, Frédéric Batteux, Jérôme Alexandre

**Affiliations:** ^1^ Paris Descartes University, Sorbonne Paris Cité, INSERM U1016, Cochin Institute, CARPEM, Paris, France; ^2^ Department of Pathology, Cochin Hospital, AP-HP, Paris, France; ^3^ Department of Genetics and Molecular Biology, Cochin Hospital, AP-HP, Paris, France; ^4^ Department of Thoracic surgery, Cochin Hospital, AP-HP, Paris, France; ^5^ Department of Gynecologic Surgery, Cochin Hospital, AP-HP, Paris, France; ^6^ Department of Medical Oncology, Cochin Hospital, AP-HP, Paris, France; ^7^ Department of Immunology, Cochin Hospital, AP-HP, Paris, France

**Keywords:** KRAS mutation, NRF2, DJ-1, primary cancer cells, non-tumorigenic cells

## Abstract

KRAS mutation, one of the most common molecular alterations observed in adult carcinomas, was reported to activate the anti-oxidant program driven by the transcription factor NRF2 (Nuclear factor-erythroid 2-related factor 2). We previously observed that the antitumoral effect of Dimethyl fumarate (DMF) is dependent of NRF2 pathway inhibition. We used *in vitro* methods to examine the effect of DMF on cell death and the activation of the NRF2/DJ-1 antioxidant pathway. We report here that DMF is preferentially cytotoxic against KRAS mutated cancer cells. This effect was observed in patient-derived cancer cell lines harbouring a G12V KRAS mutation, compared with cell lines without such a mutation. In addition, KRAS*G12V over-expression in the human Caco-2 colon cancer cell line significantly promoted DMF-induced cell death, as well as DMF-induced- reactive oxygen species (ROS) formation and -glutathione (GSH) depletion. Moreover, in contrast to malignant cells, our data confirms that the same concentration of DMF has no significant cytotoxic effects on non-tumorigenic human ARPE-19 retinal epithelial, murine 3T3 fibroblasts and primary mice bone marrow cells; but is rather associated with NRF2 activation, decreased ROS and increased GSH levels. Furthermore, DJ-1 down-regulation experiments showed that this protein does not play a protective role against NRF2 in non-tumorigenic cells, as it does in malignant ones. This, interestingly, could be at the root of the differential effect of DMF observed between malignant and non-tumorigenic cells. Our results suggest for the first time that the dependence on NRF2 observed in mutated KRAS malignant cells makes them more sensitive to the cytotoxic effect of DMF, which thus opens up new prospects for the therapeutic applications of DMF.

## INTRODUCTION

Mutations in *RAS* oncogenes are present in approximately 20% to 30% of human epithelial cancers [1], and observed in approximately 90% of pancreatic cancers, 30% to 40% of colon cancers, and 15% to 20% of lung cancers [2]. Oncogenic *KRAS* mutations mostly affect codons 12, 13, and 61; and results in the accumulation of constitutively GTP-bound RAS in cells and active downstream signaling. *KRAS* mutation has been associated with a lack of efficacy of anti-EGFR antibodies and a worsen prognosis in colorectal cancers [3]. There is therefore a need for therapies targeting *KRAS* mutated tumors. Unfortunately, RAS proteins have not yielded to any type of therapeutic attack, and, indeed, have been dismissed as “undruggable” for many years [4].

*KRAS* mutations were reported to lower the intracellular oxidative stress by activating the expression of a series of antioxidant genes via over-expression of the transcription factor NRF2 (Nuclear factor-erythroid derived 2-like 2, NFE2L2) [5]. Furthermore, genetic targeting of the NRF2 pathway was found to impair *KRAS* mutation-induced proliferation and tumorigenesis *in vivo* [5]. Thus, the inhibition of NRF2 antioxidant and cellular detoxification program may represent a therapeutic opportunity in *KRAS* mutated carcinomas.

Dimethyl fumarate (DMF), a fumaric acid derivative, has been used clinically for several years in the treatment for multiple sclerosis [6–8] and we recently identified it as a promising NRF2 axis inhibitor in cancer cells [9]. In our hands, DMF displayed concentration-dependant cytotoxicity against many cancer cell lines and this antitumoral effect was further confirmed in two mice models of colon cancer [9]. Fumarate induces the covalent modification of cysteine residues to *S* -(2-succinyl) cysteine (2SC) (termed protein succination), leading to inactivation of cysteine-rich proteins. DMF has a dual effect on the NRF2 antioxidant pathway. On one hand, it could activate the NRF2 pathway by inactivating the KEAP1 protein, which normally induces NRF2 degradation and blocks its nuclear translocation. On the other hand, DMF also inhibits the NRF2 stabilizer DJ-1, which in turn inhibits NRF2 activation, prevents its nuclear translocation, thereby inducing oxidative stress and reduced glutathione depletion; and subsequently promoting cancer cell death [9].

We hypothesize that DMF may have a preferential antitumor activity in cancers exhibiting a *KRAS* mutation. We compared the cytotoxicity; reactive oxygen species (ROS) and GSH modulations induced by DMF in several human primary tumors, with or without *KRAS* mutations and confirmed our findings by the genetic modulation of p.G12V KRAS in a Caco-2 colon cancer cell line that is not KRAS mutated. Selective toxicity of DMF to malignant cells is also a critical point in a clinical perspective. We therefore analyzed the impact of DMF on non-tumorigenic cells and compared the associated cellular events with the ones triggered in transformed malignant cells. We observed that DMF is highly cytotoxic in primary and genetically modified cancer cells harbouring KRAS mutations, whilst it was rather cytoprotective in non-tumorigenic cells. Our data support the role of NRF2/DJ1 axis in this differential effect.

## RESULTS

### DMF is especially cytotoxic in primary cancer cells harbouring a KRAS G12V mutation

**Figure 1:**
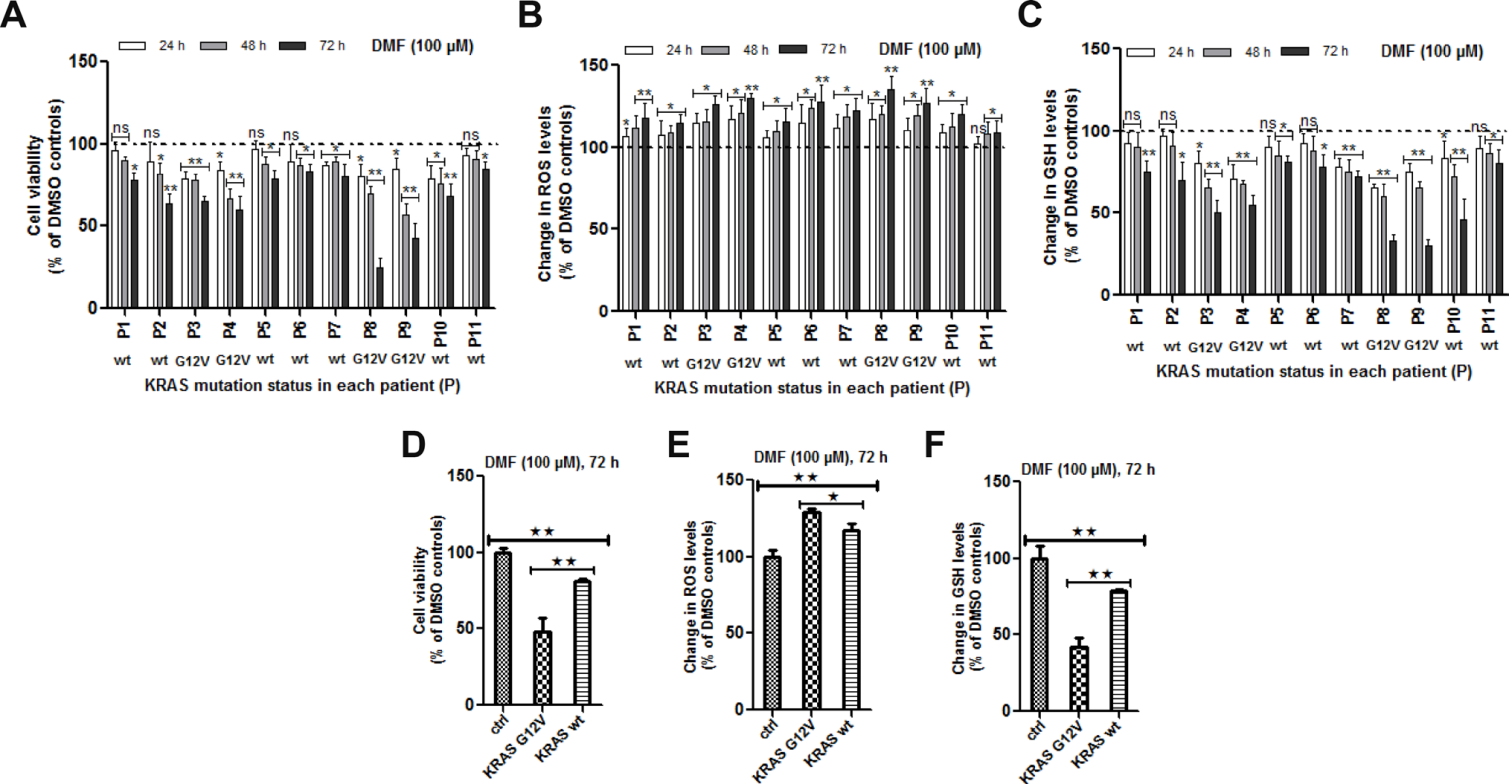
Effect of DMF on primary cells derived from patients tumors (**A**) Primary cell cultures were prepared from biopsies as previously described [13–15]. Cells were seeded in 96 well plates overnight and media changed the following day before treatment with DMSO (control) or with 100 μM DMF for 24, 48 and 72 h. A) Cell viability, (**B**) ROS production and (**C**) changes in total GSH levels were analysed as described in the “Materials and Methods” section. In (**D**–**F**), the effect of DMF in the four KRAS^*^G12V mutated patient-derived primary cancer cells (P3, P4, P8 and P9) was directly compared to another four non-KRAS^*^G12V (KRAS-wt) mutated cells (P1, P5, P6 and P11) after 72 h of treatment. This was then presented as the percentage change of the avarage for the four cell lines in each group compared to that in the other. (D) cell viability, (E) ROS production and (F) changes in GSH. In all the experiments, data are expressed as a percentage change relative to control. In each case, the mean of 3 independent experiments is shown. ^*^p < 0.05 and ^**^p < 0.01, ns = not statistically significant and P = patient (numbered from 1 to 11).

We assessed the cytotoxicity of DMF at 100 *μ*M against 11 patient-derived primary cancer cell lines of various origins: lung adenocarcinoma (*n* = 4) and squamous cell carcinoma (*n* = 1), ovarian clear cell carcinoma (*n* = 1) and high grade serous carcinoma (*n* = 2), endometrial high grade serous carcinoma (*n* = 1) and colon carcinoma (*n* = 1) (Table 1). Results shown in Figure 1A demonstrate that, in all the primary cancer cell lines, DMF was able to significantly reduce tumor viability in a time-dependent manner as well as, induce oxidative stress (Figure 1B) and GSH depletion (Figure 1C).

**Table 1:** Patients characteristics

Patient	Primary tumor (histologic type)	KRAS status	Others mutations among the ONCOMINE solid tumour DNA panel genes
P1^*^	Ovarian (HG serous ac)	WT	TP53 Arg175His
P2	Lung (scc)	WT	TP53 Arg273Leu
P3	Lung (ac)	Gly12Val	TP53 Arg158Leu
P4	Colon (ac)	Gly12Val	TP53 c.560-1G>C (splicing)
P5	Ovarian (HG serous ac)	WT	
P6	Uterine (HG serous ac)	WT	TP53 Tyr220Cys, PI3KCA His1047Arg
P7	Uterine (G1 endometrioid ac)	WT	CTNNB1 Ser33Tyr
P8	Lung (ac)	Gly12Val	TP53 Arg273Cys, PTEN 165-1G>T (splicing)
P9	Lung (ac)	Gly12Val	TP53 Arg158Pro
P10	Lung (ac)	WT	CTNNB1[Ser33Cys];[Ser37Phe], EGFR Glu746_Ser752delinsVal
P11	Ovarian (clear cell ac)	WT	PI3KCA Glu545Lys

WT: wild type, HG = high grade, ac = adenocarcinoma, scc = squamous cell carcinoma G1 = grade 1, P1^*^: patient with a BRCA1 germline mutation.^*^

To identify molecular alterations potentially associated with DMF sensitivity, several oncogenes and tumour suppressor genes most commonly mutated in adult carcinomas were sequenced in all tumours using the Oncomine solid Tumour DNA panel. *TP53* was mutated in 7 tumours but did not correlate with DMF cytotoxicity (Table 1 and Figure 1A). Four tumours harboured the KRAS*G12V mutation. The observed cytotoxic effect of DMF was significantly higher after 72 h of treatment in tumor samples with a *KRAS* mutation (mean cell survival: 48.3% and 81.3% in tumours with and without *KRAS* mutation, respectively; *p* < 0.01) after DMF treatment (Figure 1D). ROS increase and GSH depletion were also significantly higher in cells harbouring *KRAS* mutation, compared to cells with a wild type *KRAS* (ROS: 129.2% and 117.4%, respectively, of controls, *p* < 0.05; GSH level: 42.1% and 78.5%, respectively, of controls, *p* < 0.01) (Figure 1E and 1F).

### Overexpression of the KRAS*G12V mutant in Caco-2 colon cancer cells sensitised the cells to DMF cytotoxicity

**Figure 2:**
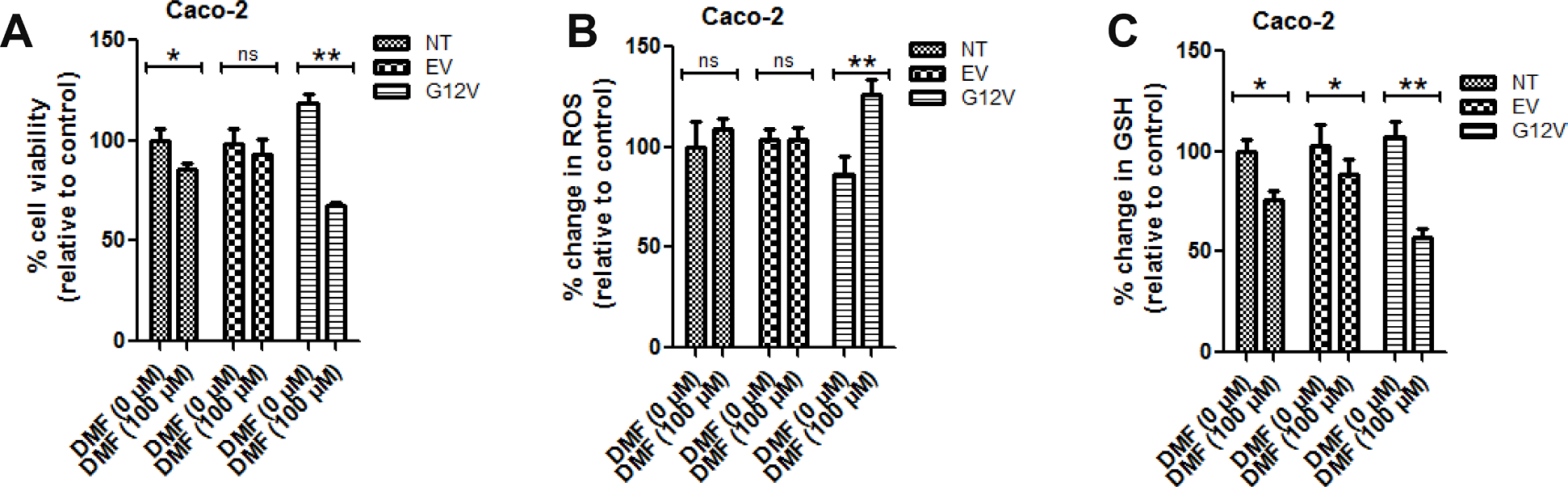
KRAS^*^G12V mutant cells are sensitive to DMF treatment Non transfected (NT), empty vector (EV) or KRAS^*^G12V-over-expressed (G12V) Caco2 cells were treated with DMSO (DMF 0 *μ*M) or 100 *μ*M DMF for 24 h. Cell viability was analysed by the Uptiblue assay method. Cells were also assayed for ROS production and GSH modulation. In all the experiments, data are expressed as a percentage change relative to control. In each case, the mean of 3 independent experiments is shown. ^*^p < 0.05 and ^**^p < 0.01.

To further gain an insight into whether DMF cytotoxicity is dependent on *KRAS* mutation, we over-expressed KRAS*G12V, a mutant form of KRAS in Caco-2 cells. In cells transfected with the empty vector, DMF did not induce any significant change in cell survival and ROS levels but only a slight decrease in GSH levels (Figure 2A–2C, respectively). By contrast, with DMF treatment, KRAS*G12V over-expression in Caco-2 cells significantly promoted DMF-induced cell death, ROS formation and GSH depletion (Figure 2A–2C, respectively). These results indicate that KRAS*G12V mutation sensitises cancer cells to DMF-induced oxidative stress and cytotoxicity.

### KRAS*G12V mutation induced NRF2/DJ-1 pathway activation

**Figure 3:**
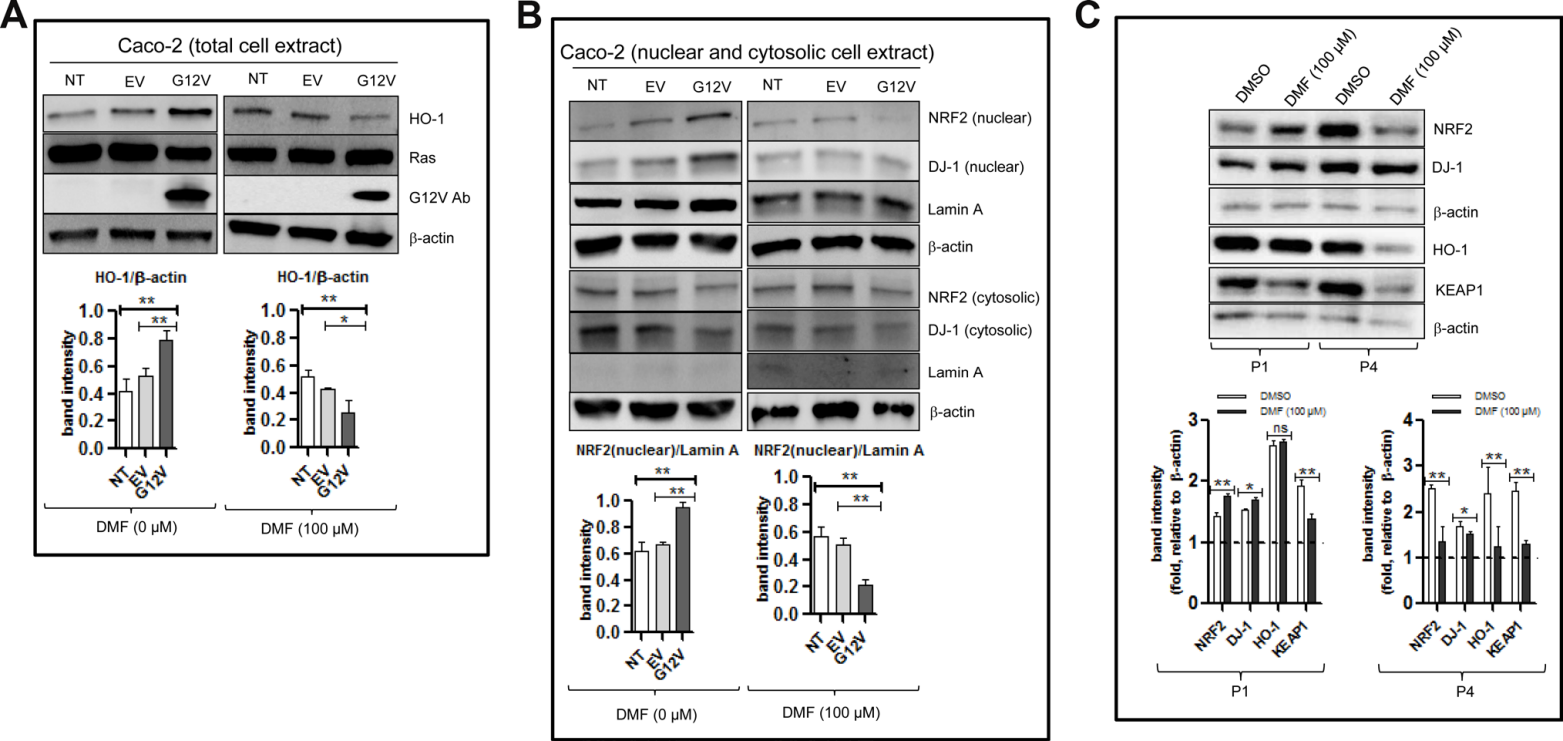
DMF activates the NRF2/DJ-1 axis in Caco-2 colon cancer cells harbouring the KRAS^*^G12V mutant Non transfected (NT), empty vector (EV) or KRAS^*^G12V-overexpressed (G12V) Caco2 cells were treated with DMSO (DMF 0 *μ*M) or 100 *μ*M DMF for 24 h. Whole cell (total cell extract) (**A**), nuclear and cytosolic lysates (**B**) were prepared. NRF2, HO-1, DJ-1, RAS and G12V protein levels were detected by Western blot. Lamin A was used as a nuclear marker, whilst β-actin was used as a loading control and in each case one representative of at least 2 independent Western blots is shown. (**C**) Primary cell cultures for patient 1 (P1) and patient 4 (P4) in Figure 1A were treatment with DMSO (control) or with 100 *μ*M DMF for 24 h. Whole cell extracts were prepared and NRF2, DJ-1, HO-1 and KEAP1 protein levels were detected by Western blot. β-actin was used as a loading control and in each case one representative of at least 2 independent Western blots is shown. ^*^p < 0.05 and ^**^p < 0.01, ns = not statistically significant.

As shown in Figure 3A (left panel), a significant increase in the protein levels of HO-1, which is a down-stream target of NRF2 was observed following KRAS*G12V over-expression; suggesting that the NRF2 pathway was activated in these cells. Indeed, compared to the empty vector, over-expression of KRAS*G12V showed an increase in the nuclear fractions of both NRF2 and DJ-1 but no significant change in their respective cytosolic protein levels (Figure 3B, left panel). In parallel, KRAS*G12V over-expression and not the empty vector reduced ROS production and promoted cell proliferation (Figure 2A, 2B respectively). KRAS*G12V over-expression was also associated with slight increase in the GSH level but the difference was not statistically significant (Figure 2C). These results suggest that the NRF2/DJ-1 antioxidant pathway activation induced by KRAS*G12V mutation allows cancer cells to decrease ROS accumulation and increase proliferation rate.

### DMF inhibits the NRF2/DJ-1 antioxidant pathway in KRAS*G12V Caco-2 cells

Treatment of KRAS*G12V Caco-2 cells with 100 *μ*M DMF for 24 h led to a significant decrease in the HO-1 level (Figure 3A, right panel) as well as in the nuclear levels of both NRF2 and DJ-1 proteins with little or no change in their respective cytosolic protein levels (Figure 3B, right panel). By contrast, HO-1 and nuclear NRF2 and DJ-1 levels were only slightly decreased after DMF treatment in Caco-2 cells with an empty vector. Furthermore, treatment with 100 *μ*M DMF for 24 h decreased HO-1, total NRF2 and total DJ-1 levels in the KRAS mutated P4; but not in the KRAS wild type P1 primary cancer cell lines (Figure 3C). The expression of KEAP1 was however, decreased in both cell lines. Taking together, these results show that DMF inhibits the NRF2/DJ-1 pathway activation induced by KRAS mutation.

### DMF is cytotoxic against cancer cells but not against non-tumorigenic cells

**Figure 4:**
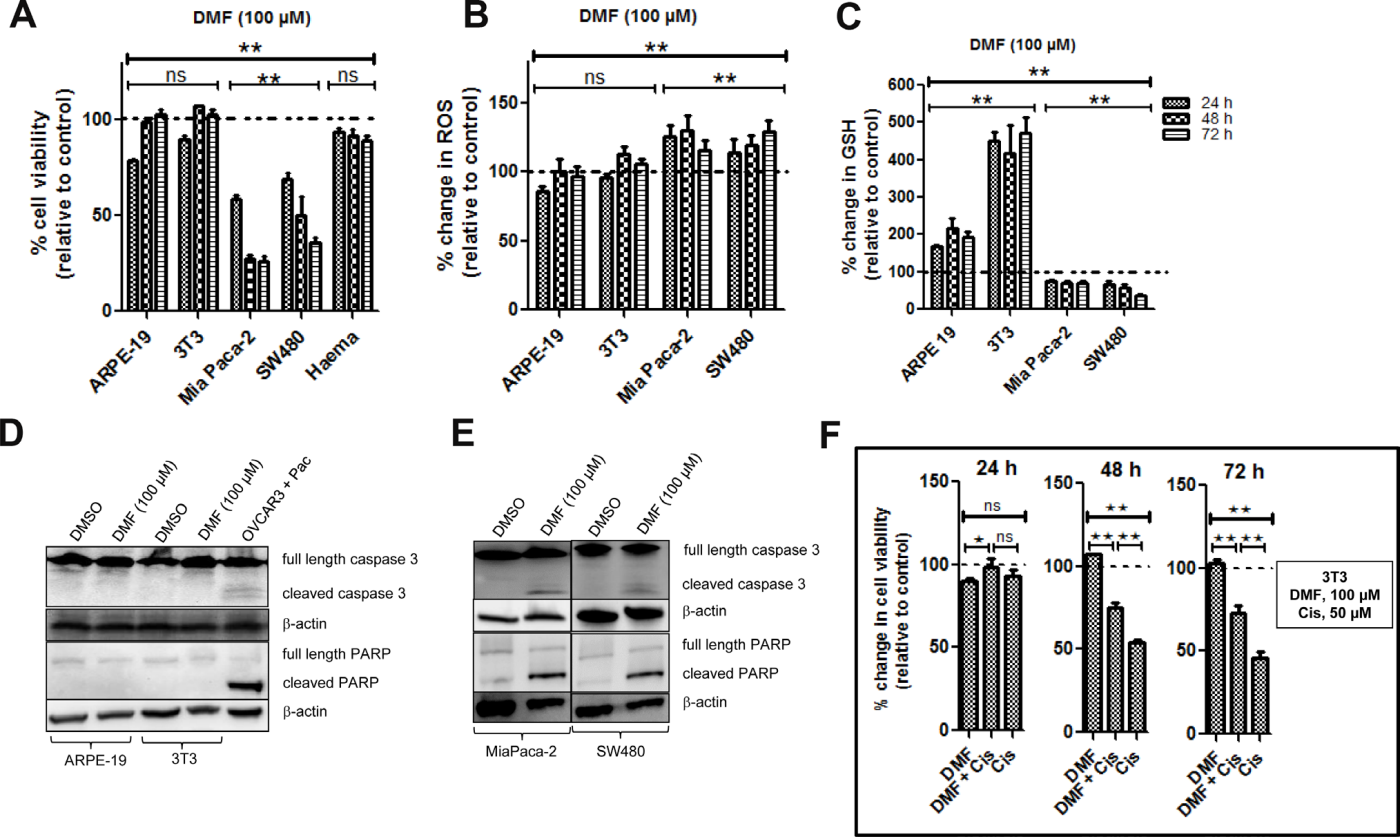
Effect of DMF on both non-tumorigenic and cancer cells Cells were seeded in 96 well plates overnight and were treated with DMSO (control = ctrl) or with 100 *μ*M DMF for 24, 48 and 72 h. (**A**) Cell viability, (**B**) ROS production and (**C**) changes in total GSH levels were analysed as described in the “Materials and Methods” section. In all the experiments, data are expressed as a percentage change relative to control. In each case, the mean of 3 independent experiments is shown. ^*^p < 0.05 and ^**^p < 0.01, ns = not statistically significant and Haema = haematopoietic cells from mouse bone marrow. (**D**) ARPE-19 and 3T3 cells were treated with DMSO or 100 *μ*M DMF for 24 h. Caspase 3 activation and cleavage of its down-stream target PARP were analysed by Western blot. (**E**) MiaPaca-2 and SW480 cells were treated with DMSO or 100 *μ*M DMF for 24 h. Caspase 3 activation and cleavage of its down-stream target PARP were analysed by Western blot as in (D). β-actin was used as a loading control. One representative of at least 3 Western blots is shown here. *DMF is cytoprotective in non-tumorigenic cells.* (**F**) 3T3 cells were seeded in 96 well plates overnight and treated the following day with DMSO (ctrl), 50 *μ*M cisplatin (Cis), 50 *μ*M cisplatin in combination with 100 *μ*M DMF for 24, 48 and 72 h. Cell viability was determined using the Uptiblue reagent. In all the experiments, data are expressed as a percentage change relative to control. In each case, the mean of 3 independent experiments is shown. ^*^p < 0.05 and ^**^p < 0.01, ns = not statistically significant.

DMF was shown as cytotoxic in a large panel of established cancer lines [9] of various origins. In order to assess a potential therapeutic index of DMF, we now asked whether DMF treatment might have a similar effect on non-tumorigenic cells. Therefore, human ARPE-19 retinal epithelial cells, murine 3T3 fibroblasts cells and primary mice bone marrow cells were treated with 100 *μ*M DMF for 24, 48 and 72 h. Cell viability was monitored by using the Uptiblue reagent. As shown in Figure 4A, there was a reduction in cell viability in both ARPE-19 and 3T3 cells by about 21 and 10%, respectively, after 24 h of treatment. Cells however recovered after 48 and 72 h. A similar effect was observed in primary mice bone marrow cells. For comparison, we treated the human MiaPaca-2 pancreatic and human SW480 colon carcinoma cell lines with 100 *μ*M DMF for the same period of time. We found a reduction in cell viability by about 42 and 31%, respectively, after 24 h, by about 73 and 50%, respectively, after 48 h and by about 74 and 64%, respectively, after 72 h.

We therefore tested the ability of DMF to induce the cleavage of both caspase 3 and PARP, as markers of apoptosis, in non-tumorigenic ARPE-19 and 3T3 cells. As shown in Figure 4 and compared to OVCAR3 paclitaxel (pac) treated cells (positive control), the cleavage of caspase 3 (Figure 4D, upper panel) along with its substrate PARP (Figure 4D, lower panel), were not induced by DMF (100 *μ*M) in ARPE-19 and 3T3 non-tumorigenic cells. We treated the MiaPaca-2 pancreatic and SW480 colon carcinoma cell lines with the same concentration of DMF and for the same period of time, we found cleavage of caspase 3 (Figure 4E, upper panel) along with PARP (Figure 4E, lower panel).

Thus, from these results, we conclude that non-tumorigenic ARPE-19 and 3T3 cells as well as bone marrow cells are more resistant against 100 *μ*M DMF treatments than the transformed malignant MiaPaca-2 and SW480 cancer cells.

### DMF modulates ROS and GSH concentrations in non-tumorigenic cells differently from that of cancer cells

As previously reported, 100 *μ*M DMF induced GSH depletion and increased oxidative stress in a time-dependent manner in the two cancer cell lines, MiaPaca-2 and SW480 (Figure 4B–4C). By contrast, in the two non- cancer cell lines ARPE-19 and 3T3, DMF applied in the same condition induces a significant increase in GSH levels (Figure 4C); while it had no significant effect on the ROS levels (Figure 4B).

Since the level of GSH is a key factor in cisplatin cytotoxicity, we decided to explore the effect of DMF on cisplatin cytotoxicity in 3T3 cells. Cells were treated with DMSO, DMF (100 *μ*M), cisplatin (50 *μ*M) or a combination of DMF and cisplatin for 24, 48 and 72 h. We observed that 100 *μ*M DMF significantly decreased the cytotoxic effect of cisplatin against 3T3 cells after 48 and 72 h of treatment (Figure 4F). In the same conditions, cytotoxicity of DMF and cisplatin was additive against cancer cells (data not shown).

Overall, DMF has an opposite effect in cancer and non-tumorigenic cells with regard to GSH antioxidant system and oxidative stress regulation. Moreover, DMF appears cytoprotective in non-tumorigenic cells.

### DMF modulates NRF2, HO-1 and DJ-1 protein expressions in non-tumorigenic cells differently from that of cancer cells

**Figure 5:**
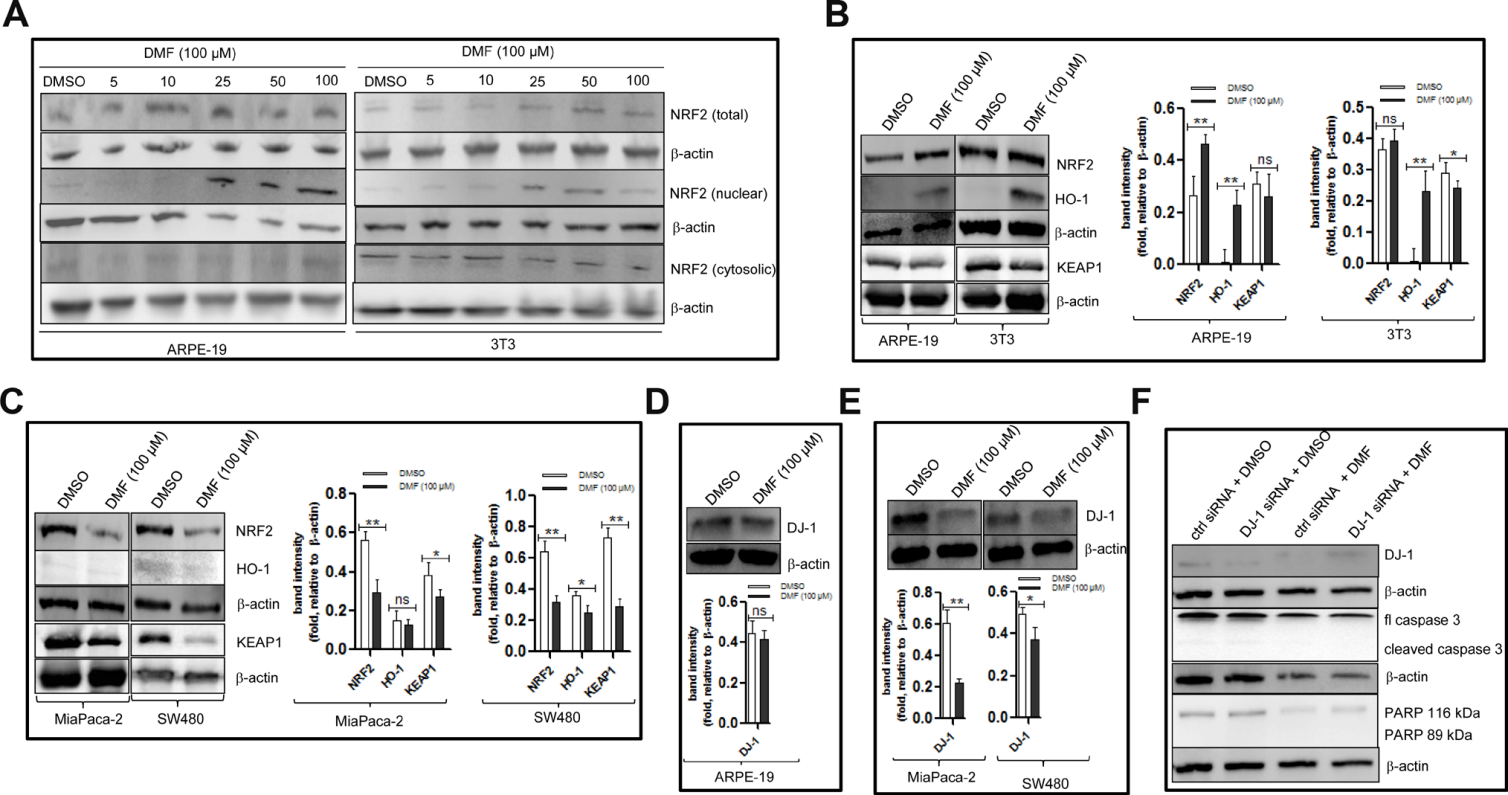
DMF modulates NRF2 and DJ-1 protein expressions in cancer cells differently to that in non-tumorigenic cells (**A**) ARPE-19 and 3T3 cells were either treated with DMSO or with increasing concentrations of DMF for 24 h. For the assessement of NRF2 sublocalisation, nuclear and cytosolic lysates were prepared and analysed on a 10% SDS-polyacrilamide gel followed by Western blottting using an anti- NRF2 specific antibody. (**B**–**E**) ARPE-19, 3T3, MiaPaca-2 and SW480 cells were seeded in 10 cm culture dishes (5 × 10^4^ cells/ml dish) overnight. The following day, medium was changed and cells were treated with DMSO or 100 *μ*M DMF for 24 h. Cell lysates were prepared and NRF2, HO-1, KEAP1 and DJ-1 protein levels were detected by Western blot. (B) NRF2, HO-1 and KEAP1 protein expression in ARPE-19 and 3T3 cells. (C) NRF2, HO-1 and KEAP1 protein expression in MiaPaca-2 and SW480 cells. (D) DJ-1 protein expression in ARPE-19 cells, and in (E) DJ-1 protein expression in MiaPaca-2 and SW480 cells. β-actin was used as a loading control. One representative of at least 3 Western blots is shown here. (**F**) ARPE-19 cells cells were transfected with the nontargeting scramble siRNA (ctrl siRNA) or DJ-1 siRNA using the NIH:OVCAR-3 Cell Avalanche^®^ Transfection Reagent as described in the “Materials and Methods” section. Twenty-four hours after transfection, the cells were treated with DMSO (ctrl) or with 100 *μ*M DMF for an additional 24 h. Whole cell lysates were prepared and analysed by Western blot using antibodies against DJ-1, caspase 3 and PARP. β-actin was used as a loading control. One representative of at least 2 independent Western blots is shown. ^*^p < 0.05 and ^**^p < 0.01, ns = not statistically significant.

**Figure 6:**
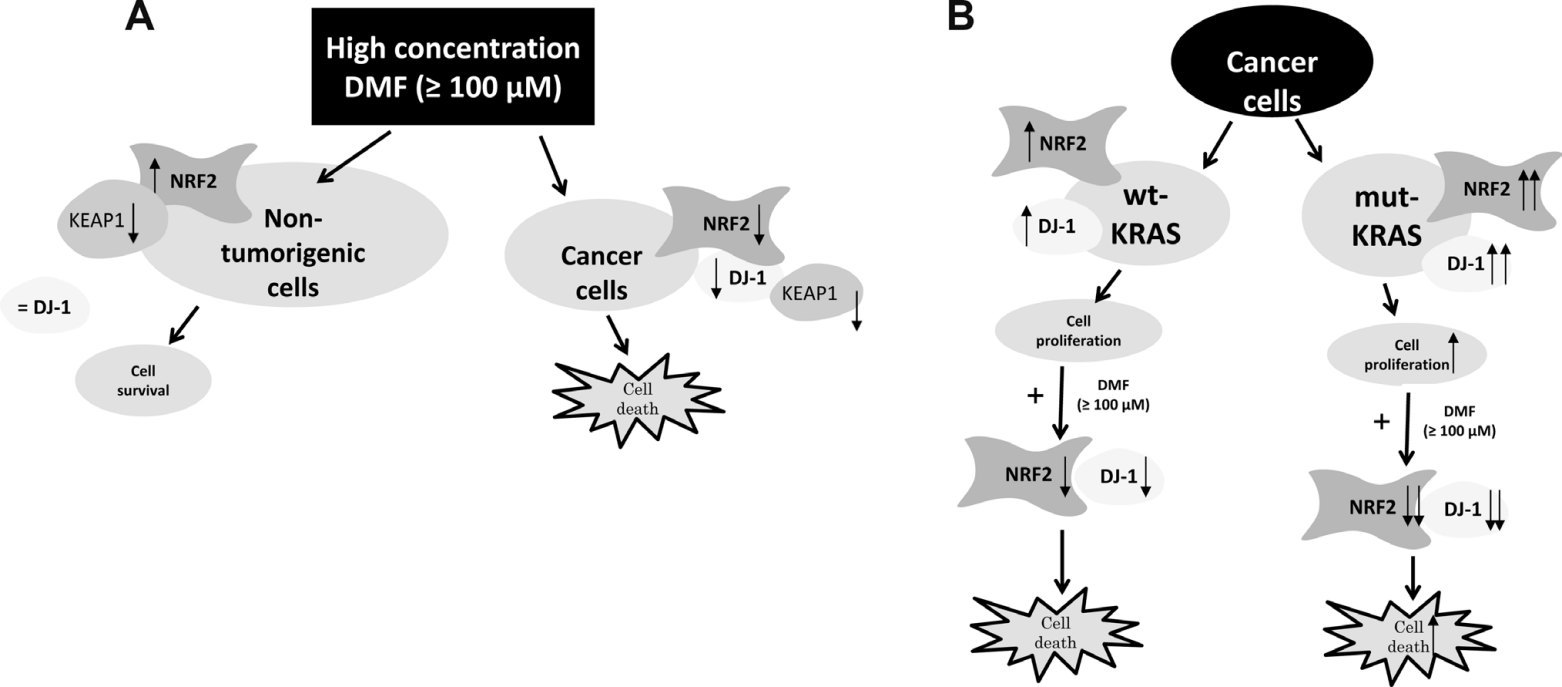
**(A)** Proposed mechanism of DMF-induced cell death in malignant and non-tumorigenic cells. (**B**) Model depicting the proposed DMF cytotoxic effect on cancer cells habouring mutant KRAS. DMF (100 *μ*M) induces disruption of the NRF2 stabiliser DJ-1, which in turn impairs NRF2 induction and transcriptional activities. It also induces ROS generation, GSH depletion, and hence, facilitates cancer cell death. This process is even more exacerbated in cancer cells habouring mutant KRAS.

We asked whether 100 *μ*M DMF will modulate the NRF2/DJ-1 axis in non-tumorigenic cells in a similar fashion than in cancer cells. As shown in Figure 5A, compared to DMSO treated cells, treatment of non-tumorigenic cells with increasing concentrations of DMF led to an increase in the protein expression levels of nuclear NRF2 level and its downstream target, HO-1 (Figure 5B). In parallel, in cancer cells, a decrease of total NRF2 and HO-1 was observed (Figure 5C). To understand the differential effect of DMF on NRF2 activation in cancer and non-tumorigenic cells, we assessed the expression of KEAP1 and DJ-1 proteins, two partners of NRF2 that are targeted by DMF. We observed that 100 *μ*M DMF significantly decreased KEAP1 protein expressions in primary (Figure 3C), established cancer cell lines (Figure 5C) and in at least one of the two non-tumorigenic cell lines (Figure 5B). Using the same protein extracts, we looked for DJ-1 protein expression in ARPE-19, MiaPaca-2 and SW480. Shown in Figure 5D, no clear change in the DJ-1 protein levels between DMSO treated and DMF treated ARPE-19 cells was observed. By contrast, a clear decrease in DJ-1 protein levels can be seen in cancer cells that were treated with DMF compared to those that were treated with DMSO (Figure 5E). This result shows that DMF modulates NRF2 and DJ-1 protein expressions in non-tumorigenic cells differently to that of cancer cells (Figure 6A). Since we previously showed that the down-regulation of endogenous DJ-1 in OVCAR3 ovarian cancer cells can make the cells more susceptible to DMF- induced apoptosis [9], we wondered if we could have a similar effect of DMF when we down-regulate endogenous DJ-1 in ARPE-19 non-tumorigenic cells. As shown in Figure 5F (upper panel), compared to control siRNA, transfection of DJ-1 siRNA in ARPE-19 cells decreases the endogenous protein levels of DJ-1. We then examined the effect of DJ-1 levels in DMF cytotoxicity. In cells treated with DMF, down-regulation of DJ-1 did not have any significant effect on apoptosis induction by DMF as evaluated by caspase 3 and PARP cleavages (Figure 5F, lower panel). Taking together, these data shows that high dose of DMF induces NRF2 activation in non-tumorigenic cells. Moreover, its cellular effect is not dependent of DJ-1 protein levels.

## DISCUSSION

Several studies have demonstrated an antitumor effect of DMF in cellular and murine models but without specifying the mechanism or identifying molecular determinants of this activity [10–16]. We report here that DMF is preferentially cytotoxic against *KRAS* mutated cancer cells. This effect was first observed in 4 patient-derived cancer cell lines (three lung and one colon adenocarcinoma) harbouring a p.G12V KRAS mutation, compared with 7 cell lines without such a mutation. In addition, KRAS*G12V over-expression in a human colon cancer cell line significantly promoted DMF-induced cell death, ROS formation and GSH depletion.

We previously explored the mechanisms of the cytotoxic and antitumor effect of DMF [9]. This effect appears to be dependent on a decrease in nuclear expression of NRF2, which is responsible for oxidative stress and glutathione depletion. NRF2 cannot be directly succinated by DMF but it is the case for DJ-1, a multifunctional protein that is encoded by the *PARK7* gene [17]. Indeed, DMF could react with the Cys106 residue of DJ-1, which is highly sensitive to oxidative stress, to form S-(2-succinyl) cysteine (2SC), leading to its inactivation [18, 19]. DJ-1 regulates NRF2-dependent antioxidant signalling by preventing its association with KEAP1 thereby, promoting its stability, nuclear translocation and activation, which provides a protective function of NRF2 with respect to the proteasome. Thus, we found that the antitumor effect of DMF is dependent on the inhibition of the DJ-1 protein.

We showed that *KRAS* activating mutation was associated with nuclear localization of NRF2 and DJ-1, and higher expression of HO-1, a downstream target of NRF2, which are hallmarks of the NRF2/DJ-1 pathway activation. By contrast, the cytosolic, inactive, fraction of NRF2 was not increased in KRAS mutated cancer cells.

KRAS mutation was shown to increase the cellular production of ROS by the mitochondrial respiratory chain [20]. The cancer-promoting effect of ROS is well known, but above a given threshold, they can be toxic to the cells. Consequently, survival and proliferation of KRAS mutated cells appear highly dependent on intracellular oxidative stress levels. Thus, KRAS mutated tumors were highly sensitive to drugs inducing oxidative stress [21, 22], while antioxidants were shown to accelerate KRAS mutated lung cancer progression in mice [23]. In this context, the activation of NRF2 pathway is an adaptive mechanism allowing KRAS mutated cells to cope with oxidative stress and increased proliferation rate [24]. Thus, mutation of KRAS creates dependence and vulnerability to the inhibition of NRF2 by DMF, which is observed to a lesser extent in non-mutated cells. However, the exact mechanisms of the NRF2/DJ-1 pathway activation associated with KRAS mutation remain to understand. A previous study has reported that DJ-1 is up-regulated in human neuroblastoma cells by the activation of the MAP kinase pathway which is downstream to KRAS protein activation [25].

A number of preclinical studies have shown a cytoprotective effect of DMF in various models of neuro-inflammation or ischaemia reperfusion injury [26–28]. However, the concentrations of DMF used in these experiments were bellow 50 *μ*M, which were generally lower than those used in cancer cells experiments. Thus, there has been no direct comparison of the effect of DMF between malignant and non-tumorigenic cells.

Here, we addressed the question of whether the various cellular signaling pathways known to be triggered by DMF in malignant cells might be also induced in non-tumorigenic cells. In contrast to malignant cells, our current data shows that the same concentration of DMF has no significant toxic effects on non-tumorigenic cells and is associated with NRF2 activation, decreased ROS levels and increased GSH levels.

While succination of DJ-1 by DMF probably inhibits DJ-1 function in both cancer and non-tumorigenic cell lines, DMF induced a decrease in DJ-1 protein level in cancer cells; this however was not apparent in non-tumorigenic cells. Above all, DJ-1 down-regulation experiments showed that DJ-1 does not play a protective role against NRF2 in non-malignant tumorigenic cells, as it does in malignant ones. Our finding is in line with a previous report in primary cortical neurons, astrocytes and *in vivo* [29]. This, interestingly, could be at the root of the differential effect of DMF observed between malignant and non-tumorigenic cells. Indeed, if DJ-1 is not necessary for NRF2 activity in non-tumorigenic cells, then the main effect of DMF remains the inactivation of the KEAP1 protein, which normally inhibits NRF2 by blocking its nuclear translocation and inducing its degradation (Figure 6).

These results suggest for the first time that the dependence on NRF2 observed in the mutated *KRAS* malignant cells makes them more sensitive to the cytotoxic effect of DMF. They thus open up prospects for the therapeutic applications of DMF.

## MATERIALS AND METHODS

### Reagents and antibodies

Protease inhibitor cocktail Complete^TM^ was obtained from Roche Diagnostics (Paris, France). The Nucleobond AX-plasmid-purification kit was from Macherey-Nagel^®^ (Dueren, Germany), NIH:OVCAR-3 Cell Avalanche^®^ Transfection Reagent was from EZ Biosystems (Maryland, USA). pBABE-Puro-KRAS*G12V was from Addgene (plasmids, # 46746). The EasySep^TM^ Human EpCAM Positive Selection Kit (cat # 18356) along with the anti-human CD326 (EpCAM), FITC-conjugated (cat # 60147FI) were from STEMCELL^TM^ Technologies (Paris, France). DJ-1 siRNA kit, antibodies against NRF2, DJ-1, HO-1, β-actin and GAPDH were all purchased from Santa Cruz Biotechnology (Heidelberg, Germany). Anti poly (ADP-ribose) polymerase (anti-PARP), anti-caspase 3, anti-Myc, anti-Lamin A, anti-G12V, anti-Ras and anti-Flag were purchased form Cell Signaling Technology (St. Quentin En Yvelines, France). Goat, mouse and rabbit secondary antibodies were all bought from Dianova (Hamburg, Germany). Paclitaxel (pac) and cisplatin (Cis) was obtained from Fresenius Kabi (Paris, France). All other chemicals (except when and where stated) were from Sigma (Saint Quentin Fallavier, France).

### Cell culture

OVCAR3 (human serous ovarian carcinoma), 3T3 (mouse fibroblast), ARPE-19 (human retinal pigment epithelium), SW480 (human colon carcinoma), Caco-2 (human colon carcinoma) and Mia Paca-2 (human pancreatic carcinoma) were obtained from the ATCC (Manassas, VA) in April 2012; where cell lines were authenticated by short tamdem repeat profiling. These cells were not reauthenticated by our laboratory but stocks were instead frozen until initiation of these studies. OVCAR-3 cells were cultured in RPMI 1640 containing insulin (10 *μ*g/ml), whilst all other cells were cultured in DMEM/Glutamax-I. All the culture media were supplemented with 10% heat inactivated Foetal Bovine Serum (FCS) and antibiotics (Life Technologies, Cergy Pontoise, France). Cells were cultured at 37° C in an atmosphere enriched with 5% CO_2_. They were passaged every 3 days and routinely tested to rule out mycoplasma infection. The seeding of cells, time of treatment and concentration of agents are shown in the figures and/or corresponding figure legends. *Isolation of haematopoietic cells from mouse bone marrow*: Haematopoietic cells were isolated from mouse bone marrow and culture as previously described [30].

### Tissue collection, cell isolation and primary cell culture

Samples were collected from 11 patients with various primary tumors (Table 1). Written informed consent from the donors for research use of tissue in this study was obtained prior to acquisition of the specimen and the study protocol was approved by the local ethics committee. Primary cell cultures were prepared from tumor samples obtained during the surgical procedure, as previously described [31]. For each sample, two populations of cells were obtained: stromal cells, and epithelial cells. The purification of these was assessed according to previous studies [32, 33]. Epithelial cells were further checked for the overexpression of Epithelial Cell Adhesion Molecules (EpCAM) using the EasySep^TM^ Human EpCAM Positive Selection Kit according to the manufacturer’s instructions. Each cell type was then cultured in its specific medium until the cells were 90% confluent and ready for treatment.

### Mutational analysis of tumor samples

Tumor samples were washed (X 2) in PBS and formalin-fixed immediately after collection from surgery. They were then paraffin embedded, and reviewed for presence, quantity, quality, and histologic type of tumor tissue by the dedicated pathologist. DNA was extracted from formalin-fixed paraffin-embedded tissue sections in selected areas containing >30% tumours cells with the Maxwell^®^ 16 *FFPE* Tissue *LEV* DNA Purification Kit (Promega). Forty ng of DNA (as measured by fluorimetry) were amplified using the Ion Oncomine™ Solid Tumour DNA Kit (ThermoFisher) according to the manufacturer’s instructions. Forty-five pM of each library was multiplexed and clonally amplified on Ion sphere particles (ISP) by emulsion PCR and enriched ISP loaded onto an Ion 318 chip with Ion Chef automate (ThermoFisher). The ISP templates were sequenced on a PGM sequencer with the Ion PGM Hi-Q Sequencing Kit according to the manufacturer’s instructions. Single nucleotide variants and small indels were detected using the Ion Reporter software (Thermofisher) version 5.2 with low stringency settings (threshold of 2%).

### Transient transfection

Transfection of cells with plasmids was performed by using the NIH: OVCAR-3 Cell Avalanche^®^ Transfection Reagent according to the manufacturer’s instructions. Briefly, cells were seeded into either a 60 mm dish (1 × 10^5^ cells) in a total volume of 2.5 ml of cell culture medium or in a 96-well plate (0.5 × 10^4^ cells) in a total volume of 100 *μ*l of cell culture. Cells were cultured overnight and were then transfected with the NIH: OVCAR-3 Cell Avalanche^®^ Transfection Reagent using a total of 0.1 *μ*g (96 well plate) or 0.25 *μ*g (6 well plate) of plasmid DNA. DJ-1 siRNA transfections were also done according to the manufacturer’s instructions. After 24 h of cultivation, the medium was replaced by a fresh one; the cells were cultured for an additional 12–18 h before treatment or then harvested for Western Blot analysis.

### Evaluation of cell viability and cell death

Cells were seeded at 0.5 × 10^4^ cells per well to a final volume of 100 *μ*l in a 96-well plate and incubated overnight. Cells were then treated with DMSO (solvent control); different concentrations of DMF or left untreated as indicated in Figures and/or corresponding Figure legends. Cell viability was monitored using the Uptiblue reagent (Interchim) as previously described [34]. After 24 h of incubation of cells with compounds, Uptiblue reagent (5%, v/v) was then added to the culture medium and fluorescence measured at 605 nm on an ELISA multi-well reader (Victor^2^, Perkin Elmer, Paris, France) after 6 h. Results are expressed as a percentage of either cell number ± SEM vs. DMSO treated cells or cell number ± SEM vs. cells in culture medium alone. Cell death was further assessed by the presence of cleaved caspase 3 and poly (ADP-ribose) polymerase (PARP).

### Intracellular ROS measurement

Cells were seeded at 1 × 10^4^ cells per well to a final volume of 100 *μ*l in a 96-well plate and incubated overnight. Cells were then treated with DMSO (solvent control); test compounds or left untreated for different time periods as indicated in Figures and/or corresponding Figure legends. ROS was then assessed spectrofluorimetrically by oxidation of 2’,7’-di-chlorodihydrofluorescein diacetate (H_2_DCFDA) as previously described [35]. Briefly, cells were washed in PBS and incubated with 100 *μ*l/well of 5 *μ*M H_2_DCFDA in PBS. ROS levels were then assayed using a fusion spectrofluorimeter (Victor^2^, Perkin Elmer, Paris, France). Fluorescence intensity was recorded every 1 h for 6 h at excitation and emission wavelengths of 485 and 530 nm, respectively. The number of cells was evaluated by the crystal violet assay and the level of ROS in each sample was calculated as follows: ROS levels (arbitrary units min^−1^ 10^4^ cells^−1^) = [Fluorescence intensity (arbitrary units) at T360 min-Fluorescence intensity (arbitrary units) at T0 min] per 60 min per number of cells as measured by the crystal violet assay. The final ROS figure (arbitrary units min^−1^ 10^4^ cells^−1^) was then expressed as a parentage relative to control. *Crystal violet assay* was done as previously described [9]. Briefly, cells were fixed and stained in 0.5% crystal violet and 20% ethanol in PBS for 30 min at room temperature. After washing twice in PBS, the stain was dissolved in 100% methanol and absorbance measured at 560 nm on an ELISA multi-well reader (Victor^2^, Perkin Elmer, Paris, France).

### Intracellular GSH measurement

Intracellular glutathione (GSH) levels were assessed as previously described [36]. Briefly, cells seeded in 96 well plates were washed in PBS and incubated with 100 *μ*l/well of 50 *μ*M monochlorobimane in PBS. GSH levels were then assayed using a fusion spectrofluorimeter. Fluorescence intensity was recorded every 1 h for 6 h at excitation and emission wavelengths of 405 and 460 nm, respectively. The number of cells was evaluated by the crystal violet assay [9], and the level of GSH in each sample was calculated as follows: GSH levels (arbitrary units min^−1^ 10^4^ cells^−1^) = [Fluorescence intensity (arbitrary units) at T360 min-Fluorescence intensity (arbitrary units) at T0 min] per 60 min per number of cells as measured by the crystal violet assay. The final GSH figure (arbitrary units min^−1^ 10^4^ cells^−1^) was then expressed as a parentage relative to control.

### Extraction of cellular proteins

Extraction of cellular proteins was performed as previously described [37]. In brief, following incubation of cells with the test compounds, cells were collected in cold PBS, pH 7.4 and centrifuged together with the cell culture medium at 4° C and 250 × *g* for 7 min. After one washing step with cold PBS, cells were lysed with 100 *μ*l of RIPA buffer (50 mM Tris-HCl, pH 8.0, 150 mM NaCl, 0.5% sodium desoxycholate, 1% Triton X-100, 0.1% sodium dodecyl sulfate (SDS)) supplemented with the protease inhibitor cocktail Complete™ according to the manufacturer’s instructions (Roche Diagnostics, Boulogne-Billancourt, France). The cell lysate was left on ice for 15 min, subjected to sonification (3 × 1 min) at 4° C and then cell debris was removed by centrifugation at 16,250 × *g* at 4° C for 30 min. Nuclear and cytosolic proteins were also prepared as previously described [38]. The protein content of the supernatant was determined according to the Bradford method using the Bio-Rad protein assay reagent (Bio-Rad, Marnes-la-Coquette, France).

### SDS-polyacrylamide gel electrophoresis and Western blot analysis

Proteins were separated on a 7.5, 10, 12.5 or 15% sodium dodecyl sulfate-polyacrylamide gel and transferred onto a polyvinylidene difluoride membrane (PVDF) by tank blotting using a transfer buffer containing 20 mM Tris- HCl, pH 8.8 and 150 mM glycine. The membrane was blocked with 5% dry milk in PBS containing 0.1% Tween-20 for 1 h at room temperature and then incubated with the specific antibody, which was diluted in PBS with 0.1% Tween-20 containing 1% dry milk powder. The membrane was washed with PBS Tween-20 containing 1% skimmed milk (3 × 10 min), before being incubated with a peroxidase-coupled secondary antibody (anti-rabbit 1:30,000 or anti-mouse 1:10,000) for 1 h at room temperature. The membrane was washed again in PBS Tween-20 (3 × 10 min). Signals were developed, visualised and quantified using the FujiFilm LAS–3000 imaging system (Velizy-Villacoublay, France).

### Statistical analysis

GraphPad Prism (GraphPad Inc., USA) software was used to analyse the data. All values are averages of at least 3 independent experiments made in triplicates, except when specified. Error bars shown in the figures represent standard error of the mean (SEM) and all results were expressed as arithmetic mean ± SEM. Differences between the experimental groups were analyzed using one-way ANOVA or student’s *t*-test (two-tail, unpaired), statistical significant differences were shown as follows: ^**^p < 0.01 or ^*^p < 0.05.
